# Lung ultrasound in neonates and children with cardiac diseases with focus on post cardiac surgical period: time for systematic use—an expert opinion report by the Association for European Paediatric and Congenital Cardiology Imaging Working Group

**DOI:** 10.1093/ehjimp/qyae134

**Published:** 2025-01-13

**Authors:** Massimiliano Cantinotti, Giovanni Di Salvo, Inga Voges, Francesca Raimondi, Gerald Greil, Almudena Ortiz Garrido, Heynric B Grotenhuis, Colin J McMahon

**Affiliations:** Fondazione CNR-Regione Toscana G. Monasterio (FTGM) , Ospedale del Cuore, via Aurelia Sud, Massa, Pisa 54100, Italy; Paediatric Cardiology and Congenital Heart Disease, Woman and Children’s Health Department, University of Padua, Experimental Cardiology, Paediatric Research Institute (IRP), Padua, Italy; Department for Congenital Cardiology and Paediatric Cardiology, University Hospital SchleswigHolstein, Campus Kiel, Kiel, Germany; German Center for Cardiovascular Research (DZHK), Partner Site Hamburg/Greifswald/Kiel/Lübeck, Kiel, Germany; Ospedale papa Giovanni XXIII, Bergamo, Italy; Division Paediatric Cardiology, UT Southwestern, Dallas, TX, USA; Department Paediatric Cardiology, Malaga, Andalucia, Spain; Department Paediatric Cardiology, Wilhelmina Children’s Hospital/UMCU, Utrecht, The Netherlands; Department Paediatric Cardiology, Wilhelmina Children’s Hospital/UMCU, Utrecht, The Netherlands; Department Paediatric Cardiology, Children’s Health Ireland at Crumlin, Dublin 12, University School of Medicine, University College, Dublin 4, Ireland

**Keywords:** echocardiography, children, congenital heart disease, reporting, formats, scores, lung, ultrasound

## Abstract

**Background:**

Despite lung ultrasound (LUS) gaining consensus for the diagnosis of pulmonary complication in paediatric acute care setting and in adult cardiology, its use in paediatric cardiology remains limited.

**Aim:**

The aim of the present investigation is to provide an expert opinion on the applications of LUS in neonates and children with congenital heart disease, with a special focus on the post-surgical period.

**Methods and Results:**

A complete guide for identification of landmarks and major signs (A and B lines) and their characteristics is provided. Diagnostic criteria, tips, and tricks for the diagnosis, and differential diagnosis of common pulmonary diseases such as pleural effusion, pneumonia, and consolidation are provided. To perform diagnosis of pneumothorax is illustrated. Applications of LUS for evaluation of hemidiaphragm motility and for a comprehensive assessment of retrosternal area are also discussed. The use of LUS for guidance of minor, common interventional procedures such as lung recruitment and drainage insertion is also described. The report also highlights current gaps of knowledge, including the difficulty for quantitative estimation of pleural effusion and atelectasis, and future prospective.

**Conclusion:**

There is sufficient evidence to support a systematic use of LUS for the diagnosis and follow-up of neonates and children with cardiac disease especially those undergoing paediatric cardiac surgery. LUS is an easy, accurate, fast, cheap, and radiation-free tool that should become a routine in daily practice.

## Graphical Abstract

**Graphical abstract:**
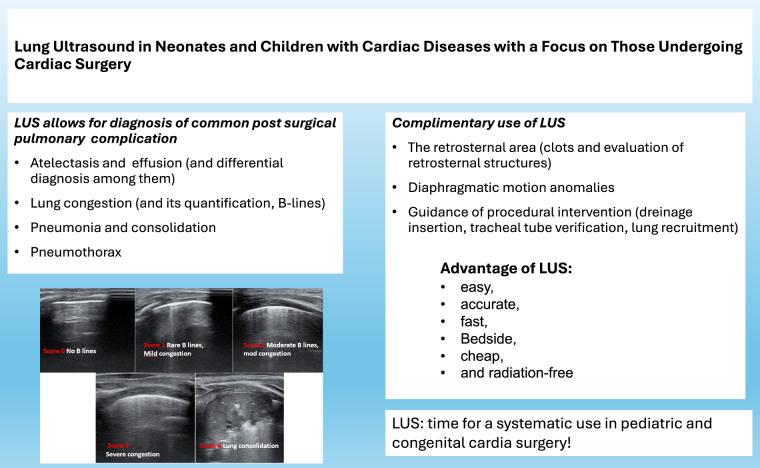

## Introduction

Lung ultrasound (LUS) has gained consensus for diagnosis of pulmonary diseases in the acute paediatric setting^1–6^ and in adults with chronic and acute heart failure.^2^ Neonates and children with cardiac diseases, particularly those undergoing cardiac surgery, represent an ideal field of application for LUS since pulmonary complications (e.g. atelectasis, effusion, lung congestion, pneumonia, pneumothorax, obstructive pulmonary disease, and diaphragmatic motion anomalies) are very common.^7–9^ Compared to traditional chest radiography, LUS is a radiation-free technique that offers several advantages, including the possibility at the bedside for a quick monitoring of lung disease progression and response to medical therapy (i.e. diuretics) and physiotherapy.^7–9^ Furthermore, LUS allows for differentiation of many common pulmonary complications after paediatric surgery (e.g. effusion vs. atelectasis), requiring different therapeutic approaches.^1–6^ It has been demonstrated that the systematic use of LUS in paediatric cardiac surgery settings may reduce the use of chest radiograph and individual radiation dose,^10^ which is particularly important for neonates and children.

Compared to other paediatric settings,^3–6^ the role of LUS in paediatric cardiology has remained limited,^7–9^ probably due to the traditional practice of relying primarily on chest radiography.

The aims of this manuscript are to:

- Identify settings where LUS should be employed in neonates and children with cardiac disease.
- Propose a basic standard for LUS examination in paediatric cardiology.
- Identify basic elements for diagnosis of major pulmonary disease in neonates and children with cardiac disease with a focus on those after paediatric cardiac surgery.
- Review the potential and limitations of LUS for diagnosing pulmonary complications after paediatric cardiac surgery.

## General principles

### LUS examination protocols

**Figure 1:**
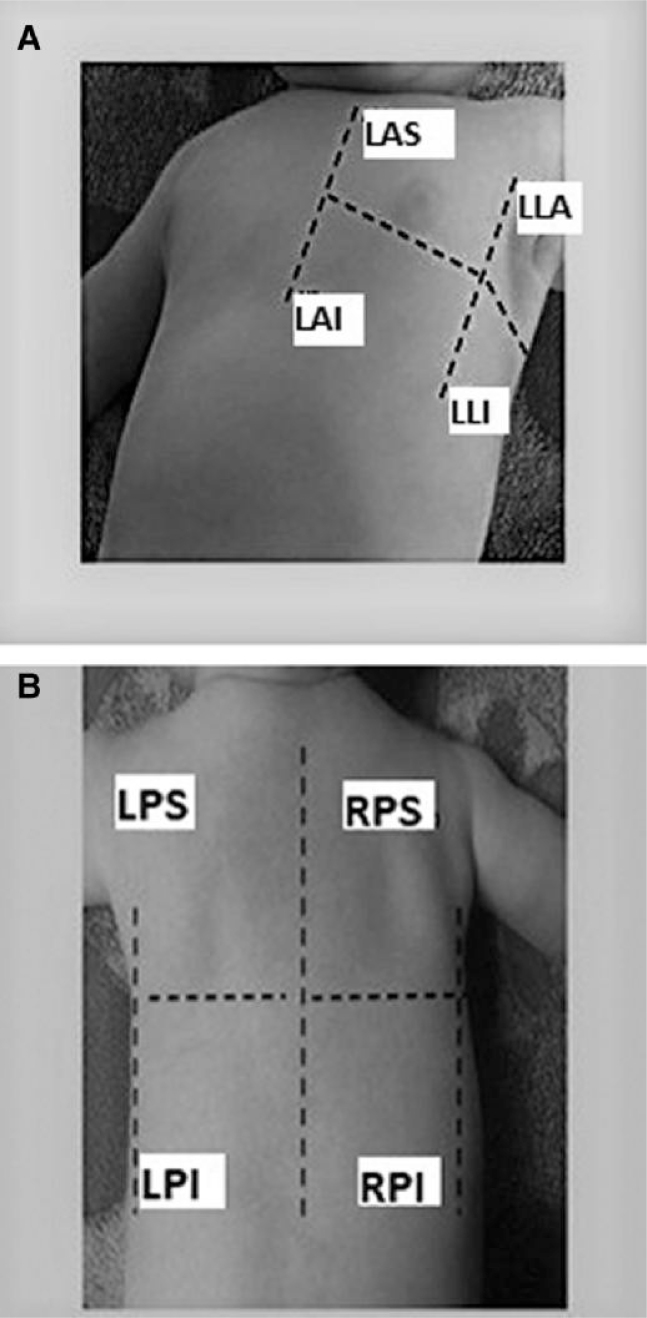
Six segments’ score. Each hemithorax is divided into three major quadrants (anterior, lateral, and posterior). Each quadrant is further subdivided into the upper and the lower half. (*A*) Anterior al lateral quadrants. (*B*) Posterior quadrants. LAS, left anterior superior; LAI, left anterior inferior; LPS, left posterior superior; LLA, left lateral anterior; LLI, left lateral inferior; LPI, left posterior inferior; RPS, right posterior superior; RPI, right posterior inferior.

LUS examinations may be performed with either phased-array probes or with linear/convex probes. In neonates and children, linear and convex probes are preferred, since they offer a quick and comprehensive view of the entire lung field. According to standardized adults’ protocols,^11^ LUS examination should be performed in different views and positions and include evaluation of different pulmonary areas. A complete LUS examination should include the evaluation of three major areas (anterior, lateral, and posterior) for each hemithorax delineated by the para-sternal, anterior axillary, and posterior axillary line, respectively.^11–13^ Each area can be further divided into an upper and lower half, creating four to six different quadrants for each hemithorax, namely anterior superior, anterior inferior, lateral superior, lateral inferior, posterior superior, and posterior inferior^11^ (*Figure 1*).

The posterior view is of paramount importance in neonates and children after cardiac surgery. Examinations in the posterior areas were found to be more sensitive than anterior and lateral in the diagnosis of effusion or atelectasis,^12^ the two most common pulmonary complications after cardiac surgery. When performing posterior examination, it is important to start delineating the diaphragm, which is the fundamental landmark to differentiate the lung from the liver.^12,13^ Once the base of the lung has been delineated, the entire lung may be easily and quickly scanned with a continuous sweep up to the apex.^12,13^ The posterior view, however, may not be feasible to examine by LUS in all the subjects. The posterior examination may be precluded in unstable children, those with an open sternum, poorly cooperative children, or children with poor mobilization. In studies on paediatric cardiac surgery populations,^12,13^ the posterior area was precluded in 7%, while the anterior area could not be assessed in 11% due to bandages and medications covering a substantial part of the hemithorax. In contrast, LUS examination was almost always feasible in the lateral area (e.g. feasibility 98%), despite the frequent presence of drainage tubes and other physical impediments.^12,13^

#### Key points

- LUS may be performed either with phased-array or linear/convex probes, with the latter to be preferred especially in neonates, infants, and young children.
- For each hemithorax, two or three major areas (anterior, lateral, and sometimes posterior) need to be evaluated.
- Posterior examination (when feasible) is more sensitive for diagnosis of pulmonary complications.

### When and who should perform LUS

LUS may be performed either as a completion of routine echocardiographic examination or as a separate examination.^11–13^ LUS may be used to monitor pulmonary complications in children with cardiac disease and their response to medical therapy and/or physiotherapeutic treatment.^11–13^ Regular LUS examination in intensive care units may be helpful to monitor pulmonary complication after paediatric cardiac surgery. LUS examination however should be repeated at any time, in case of clinical variations (e.g. desaturation, hyperventilation, and acute cardiac failure).^10^ In the ward, time intervals for LUS examination may vary according to the cardiac disease and clinical condition. In uncomplicated cases, LUS may be performed in addition to routine echocardiographic examination (e.g. post electrode removal and before discharge), while in patients who are more prone to develop pleural effusions (e.g. univentricular heart after stages II and III of Fontan palliation and tetralogy of Fallot after correction), more frequent LUS examinations may be helpful to guide diuretic therapy and fluid balance.^10^ LUS may be also used to guide interventional procedures such as drainage insertion and attempts at resolving atelectasis by manual lung recruitments with positive airway pressure manoeuvres.^11–13^

LUS examination is a very easy technique requiring basic knowledge of ultrasound principles, ultrasound equipment, and a limited period of training.^11–13^ There is a need for a short training period for a paediatric cardiologist expert in echocardiography, but even for medical/paramedical staff with basic capabilities to interpret echo images.^11–13^ LUS should be performed by the cardiologist who performs routine postoperative echocardiographic examination, as a completion of echocardiography.^11–15^ LUS, however, may also be performed by any physician who oversees the patient (e.g. anaesthetist, cardiologist, and surgeon).^11–15^ Theoretically, LUS may also be performed by allied healthcare professionals,^14,15^ including nurses^15^ and physiotherapists,^14^ to guide treatment and monitor results.^14^ LUS for instance may be used before and after physiotherapeutic treatment to monitor patients with atelectasis.^14,15^

#### Key points

- LUS should be performed during routine echocardiographic examination as a completion of routine examinations.
- Time intervals for LUS examination repetition post paediatric cardiac surgery should vary according to the cardiac disease (and its correction/palliation) and to the clinical condition.
- LUS may be performed to monitor interventional procedures (e.g. drainage insertion and atelectasis resolution).
- LUS requires a limited knowledge of ultrasound and requires a short training period for a paediatric cardiologist.
- LUS may be performed by all professionals who participated in the patient’s care, including allied healthcare professionals.

## Diagnosis of lung disease

### Common findings

#### B lines

B lines are the sonographic indirect sign (e.g. artefacts) of partial de-aeration of the lung parenchyma.^11^ B lines may be due to extravascular fluid accumulation or to a de-aeration of other nature. B lines are often seen at the border of areas of de-aeration (such as atelectasis).^11^ B lines are almost universally present in neonates and children with congenital heart disease (CHD) after paediatric cardiac surgery either due to extravascular fluid accumulation [particularly after cardiopulmonary bypass (CPB)] or to areas of atelectasis involving the lung.^7–9^ B lines due to extravascular fluid accumulation and de-aeration from chronic hypoxia or recovery from atelectasis are also present in children with CHD with increased pulmonary blood flow, ventricular dysfunction, valve defects, etc.^7–9^ In neonates and children with heart failure due to CHD with pulmonary overcirculation (e.g. left-to-right or bidirectional shunt), LUS held a high sensitivity, specificity, and diagnostic accuracy (94, 96, and 95%, respectively) for the assessment of lung congestion from pulmonary overflow compared to computed tomography (CT).^16,17^

**Figure 2:**
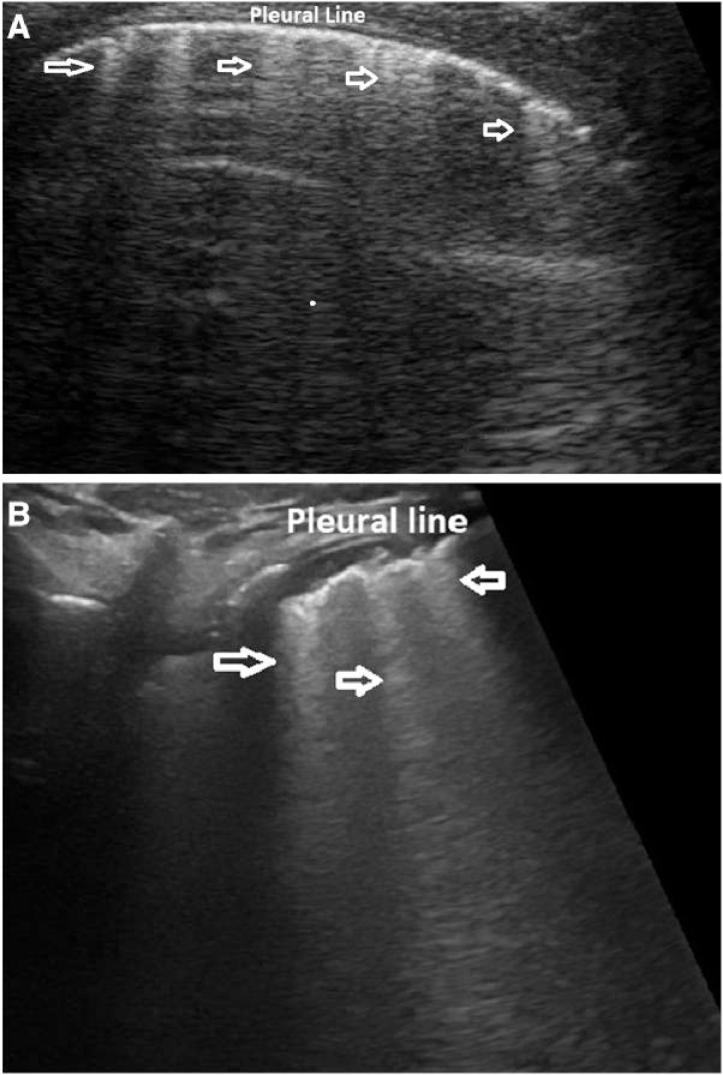
(*A*) Regularly distributed, thin, cardiogenic B lines (arrows). (*B*) Irregular, patchy B lines (arrows) associated with pneumonia.

It can be difficult to differentiate B lines due to cardiogenic lung congestion from other forms of lung de-aeration, although some characteristics may prove helpful. In cardiogenic lung congestion, B lines due to cardiogenic lung congestion (e.g. extravascular fluid accumulation) are uniformly present on either hemithorax with a gravity-dependent distribution and are typically thin with regular pleural lines^11,18^ (*Figure 2*; *Video 1*). Irregular, patchy B line distribution, often with irregular pleural lines, is more commonly seen in other forms of lung disease [e.g. atelectasis, pneumonia, acute respiratory distress syndrome (ARDS), or pulmonary fibrosis]^11,18^ (*Figure 2*).

##### Classification of lung congestion in children

**Figure 3:**
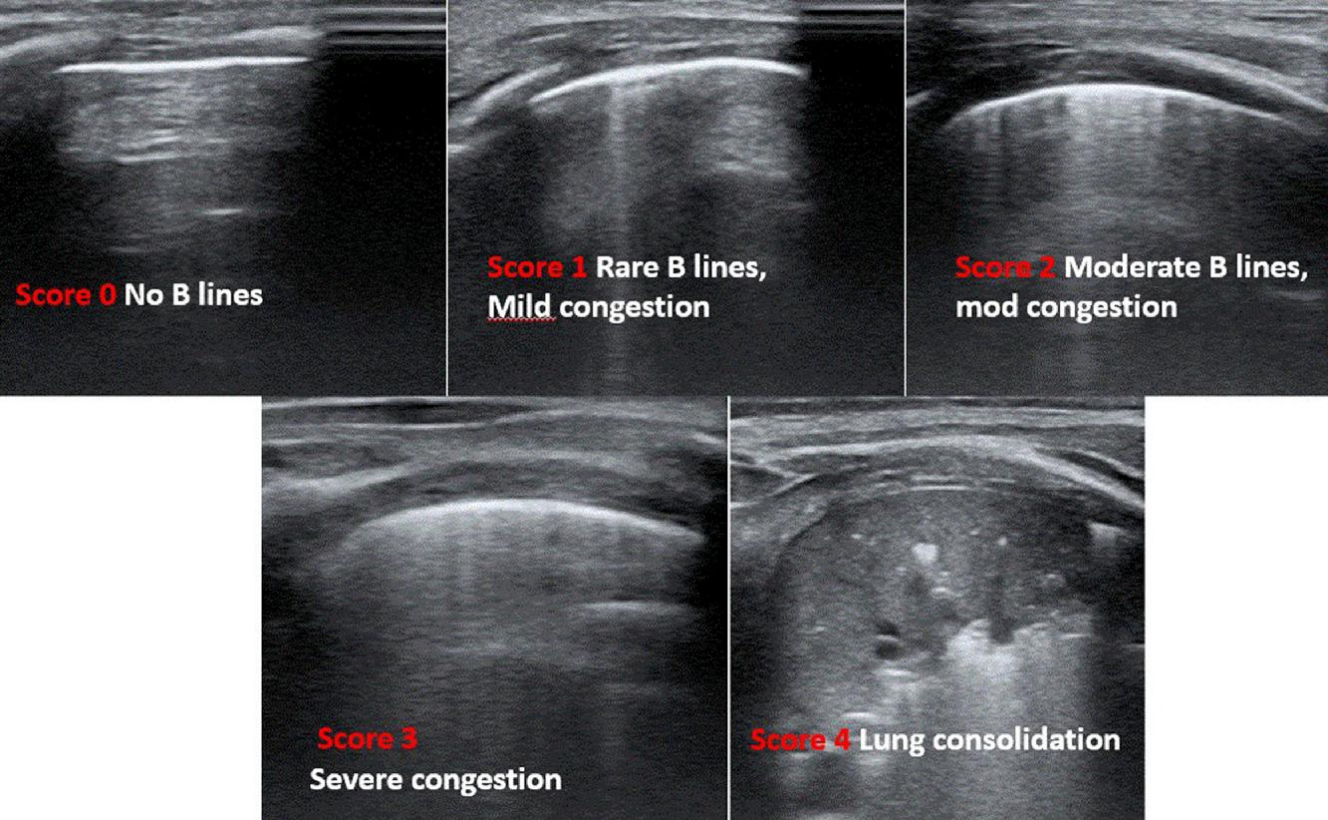
An example of semiquantitative LUS score that we have recently validated.

Lung congestion degree may be classified in different ways.^13,16–18^ In adults, B lines are counted in each scanning area, and a score can be assigned for either single quadrants or for the entire hemithorax, the latter quantified by summing partial scores for each single scanning area.^18,19^ Scores to classify lung involvement in adults with heart failure are either the sum of B lines in each area or the number of areas with more than three B lines^18^; lung involvement is commonly classified into four categories (none, mild, moderate, and severe).^18,19^ Simplified scores, either qualitative or semiquantitative scores, have been adopted in children. Some authors have proposed semiquantitative scores^13,17^ (*Table 1*). Wu *et al.*^16^ defined four patterns from normal (absence of B lines) to severe congestion (e.g. more than seven or coalescent B lines from the base to the apex without spared area). Cantinotti *et al.*^12,13^ suggested a score that is the sum of B lines in different scanning areas for a single hemithorax. The degree of lung congestion was classified from I (trivial-none, LUS score = 0–6) to severe (LUS score >24). Vitale *et al.*^7,21^ have proposed a qualitative score identifying three different patterns: (i) white lung, defined as the presence of confluent B lines in two or more of the four areas; (ii) the prevalence of B lines in two or more areas; and (iii) the prevalence of A lines or no significant congestion/normal lung (*Figure 3*).

**Table 1:** Major semiquantitative scores for lung congestion classification in children

Authors	Classifications
Wu *et al.*^16^	Normal: A lines Mild: fewer than 3 B lines in 2 rib spaces with spared areas Moderate: between 3 and 7 B lines between 2 rib spaces Severe: more than 7 or coalescent B lines from the base to the apex without spared area
Cantinotti *et al.*^12,13^	Trivial-none (LUS score = 0–6) Mild (LUS score = 6–12) Moderate (LUS score = 13–24) Severe (LUS score >24)
Vitale *et al.*^15,20^	Type 1: full hyperechoic image of the lung fields or ‘white lung’. Type 2: prevalence of B lines, that is, vertical, comet-tail artefacts. Type 3: predominance of A lines

LUS, lung ultrasound.

The degree of lung congestion may have prognostic potential in children after cardiac surgery for congenital heart disease.^12,20,21^ Lung congestion at 12–36 h after surgery correlated with longer CPB and cross clamp times,^1^ longer need of mechanical ventilation,^12,21^ and lengthened stay in ICU.^12,21^

###### Key points

- LUS should be used to identify and quantify the presence of B lines.
- Scores may be employed to quantify B lines in neonates and children. These scores differ from those employed in adults and may be either semiquantitative or qualitative.
- B lines due to extravascular fluid accumulation are regular, uniformly present on either hemithorax with a gravity-dependent distribution.
- B lines due to other forms of lung de-aeration (e.g. atelectasis, pneumonia, ARDS, and pulmonary fibrosis) are irregular, patchy distributions, often associated with irregular pleural lines.

###### Lack of knowledge

- Uniform scores for B line severity classification are currently lacking.
- Automatic and semi-automatic methods for B line quantification are also lacking.

#### Pleural effusion

**Figure 4:**
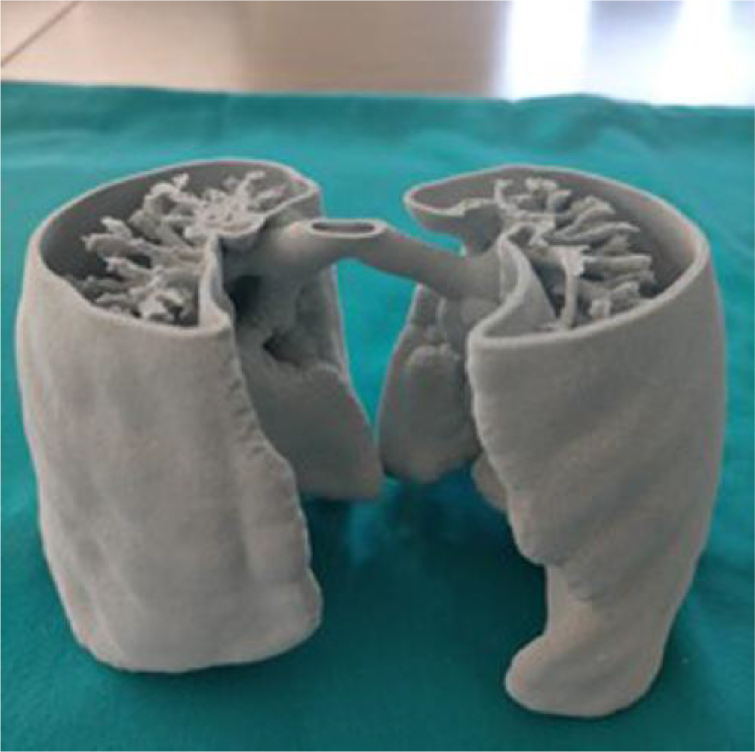
3D printing from a CT scan of lungs of healthy children. The irregular geometric figure can be appreciated.

Evaluation of pleural effusion is an established application of LUS for many years now (*Figure 4*). Compared to CT, which remains the diagnostic gold standard, but is invasive, time consuming, expensive, and exposes the child to radiation,^10,22^ LUS demonstrates a high sensitivity and specificity (93%) for the diagnosis of pleural effusion. Compared to chest X-ray, LUS offers the advantage in the differential diagnosis of pleural effusion vs. atelectasis and the capability to define the nature of post-surgical pleural effusion.^7,9,22–29^ For instance, while anechoic effusion may be either a transudate or an exudate, the presence of internal echoes is highly suggestive of an exudate (such as pneumonia, pulmonary embolism, and tuberculosis) or a haemothorax or a chylothorax.^29,30^ LUS is an easy, fast, radiation-free technique that may be employed at bedside to monitor the effusion progression and/or response to medical therapy. LUS furthermore may be used to guide thoracentesis and to monitor its results.^10^ Despite the evaluation of pleural effusion being commonly performed even by medical/paramedical staff not expert in LUS, many aspects remain unclear. Posterior area examination is more sensitive than anterior and lateral in the diagnosis of effusion^12^; however, how the examination should be performed (sitting or supine position) has not been standardized yet. The sitting position may offer an estimate closer to chest X-ray, but it may be not feasible in unstable non-cooperating patients.^12^

How to quantify the extent of pleural effusion has not yet been standardized. Quantification of pleural effusion by LUS is often qualitative, and a classification that uses the terms mild, moderate, or severe is used. In adults, various algorithms, each using different projections and measurement methods, have been proposed for pleural effusion quantification,^23–27^ though none of these have been validated for infants and children (*Table 2*). The difficult geometry of the lung makes the search for mathematical formulas challenging in this circumstance (*Figures 3* and *4*, Supplementary data online, *Figure S1* and *Videos S1* and *S2*).

**Table 2:** Major studies proposing formula for pleural effusion quantification

Authors	Population	Protocol of examination	Formula	
Remérand *et al.*,^24^ France	58 (45 M) Age 58 ± 17 years	Supine Transverse views positioning the transducer in each IS. The transducer slipped between the patient’s back and mattress. The lower and upper IS where PE was detected were drawn on the patient’s skin. PE length measured in paravertebral regions between the apical and caudal limits. Cross-sectional area measured at the midlength of PE.	PEV (mL) = ACT (cm^2^) × LCT (mm)	
*Usta et al.*,^26^ Germany	135 (90 M) Age 60 (45–67) years	Sitting The transducer was moved in a cranial direction in the mid-scapular line. PE diameter: maximal distance between midheight of the diaphragm and visceral pleura.	PEV (mL) = D (mm) × 16	
Balik *et al.*,^25^ Czech Republic	81 (47 M) m. ventilated patients Age 60 ± 15 years	SUPINE The transducer was moved in the cranial direction in the posterior axillary line. PE diameter: maximal distance between parietal and visceral pleura at the lung base.	PEV (mL) = 20 × Sep (mm)	
Eibenberger *et al.*,^27^ Austria	51 (21 M) Age 28–82 years	Sitting Latero-dorsal wall of the chest. PE diameter: the maximal perpendicular distance between the posterior wall of the lung and the posterior chest wall.	D (mm) PEV (mL) 0 10 20 30 40 50 > 60	PEV (mL) 0–90 50–300 150–310 160–660 490–1670 650–1840 950–2510
Vignon *et al.*,^23^ France	97 (61 M) Age 59 ± 20 years	Supine From the base to the apex of the chest, along the dorso-lateral part of the chest wall, as far as possible posterior between the mattress and the patient’s back without lifting the hemithorax. PE diameter: the maximal distance from the leading edge of the dependent surface of the lung to the trailing edge of the posterior chest wall, on transverse views of pleural spaces. Measurements were made at the base and at the apex of the pleural space.	D > 45 mm at the RTB D > 50 mm at the LTB base predicted a PEV ≥ 800 mL sensitivity of 94 and 100% and specificity of 76 and 67%, respectively.	

Typically, the interpleural distance was greater at end-expiration in ventilated patients and on inspiration in spontaneously breathing patients.

ACT, pleural effusion cross-sectional area; EE, end-expiration; EI, end-inspiration; IS, intercostal space; LCT, pleural effusion length; LTB, left thoracic base; M, males; m., mechanical; PEV, pleural effusion volume; RTB, right thoracic base; Sep, separation; V, volume; D, diameter; PE, pleural effusion; BMI, body mass index.

##### Key points

- The use of LUS is recommended for diagnosis and follow-up of pleural effusion.
- LUS can help to differentiate between effusion and atelectasis, and to define the nature of post-surgical pleural effusion.

##### Gaps of knowledge

- No accurate mathematical formula exists to correctly quantify size of the effusion, and formulas derived for adults are poorly applicable to the paediatric population.
- Systems to classify effusion severity (at different ages and body size) are lacking.

#### Atelectasis, pneumonia, consolidations, and others

Compared to chest X-ray, LUS allows for differentiating among possible diagnoses among atelectasis, effusion, and different forms of consolidation and masses (e.g. pneumonia, infarction, contusion from thoracic trauma, and primary or metastatic cancer clots).^12,29,30^

##### Atelectasis

Atelectasis is very common in children with CHD, especially those confined to bed and those requiring anaesthesia (e.g. about 68–100% after different types of surgery)^30–34^ and is the most common pulmonary complication (together with effusion) after paediatric surgery. The aetiology of atelectasis after cardiac surgery is multifactorial including surgical compression, CPB, consequences on the lung of cardiac defect, and inappropriate ventilation. Atelectasis after cardiac surgery more commonly occurs in the inferior–posterior region (60–92.7%) than in the anterior (5–20.7%) or lateral regions (5–13.8%).^12,34^

As for pleural effusion, how to quantify the extent of atelectasis has not yet been standardized. As a result, quantification of atelectasis by LUS is qualitative, classified as mild, moderate, or severe (Supplementary data online, *Figure S2*).

##### Pneumonia

In a recent^35^ large meta-analysis that evaluated the value of LUS in 2470 children with pneumonia, LUS demonstrated a high sensitivity (0.95; 95% CI: 0.94–0.96) and specificity (0.90; 95% CI: 0.87–0.92), and diagnostic odds ratio (137.49; 95% CI: 60.21–313.98) for the diagnosis of pneumonia in the paediatric age group.

Today, LUS is widely used in NICU for the diagnosis of pneumonia.^3–6,35^ Diagnostic criteria for the diagnosis of pneumonia by LUS include the presence of consolidation (area with no A lines), dynamic air bronchograms, alveolar–interstitial syndrome (increased B lines surrounding consolidated area), presence of blood flow on colour Doppler sonography, and pleural effusion^3–6,35^ (Supplementary data online, *Figure S3*).

###### Key points

- LUS allows for diagnosis of atelectasis and for differential diagnosis among different forms of consolidation and masses (e.g. pneumonia, infarction, contusion from thoracic trauma, and primary or metastatic cancer clots).
- The use of LUS has a high sensitivity and specificity in the diagnosis of pneumonia in children.

###### Gaps of knowledge

- No accurate mathematical formula exists to correctly quantify the size of the atelectasis.
- Systems to classify atelectasis severity (at different ages and body size) are lacking.

#### Pneumothorax

**Figure 5:**
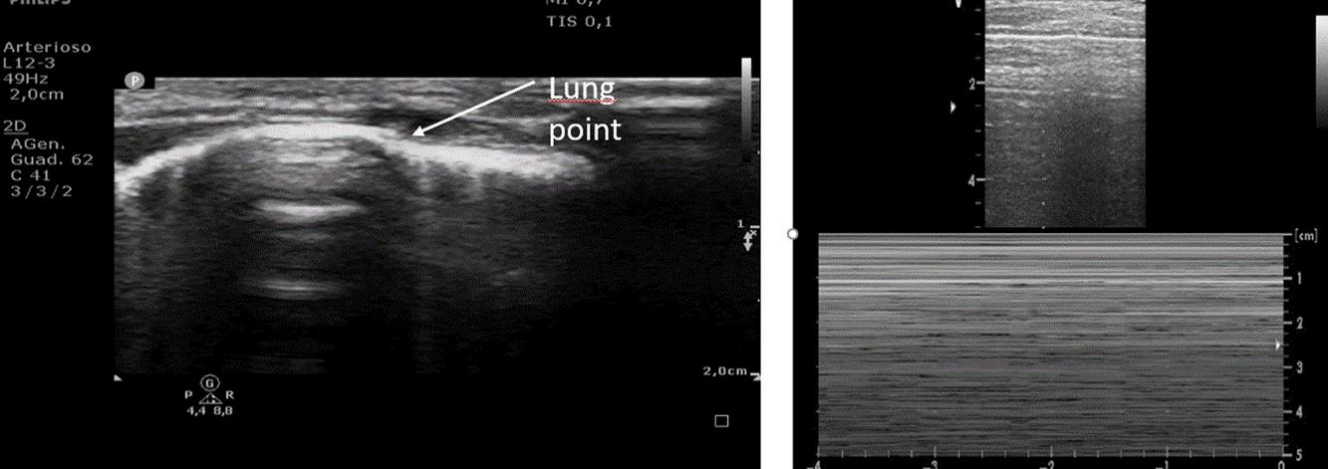
Diagnosis of pneumothorax. The lung point (e.g. the point where the pleura stops its movement) is highlighted on the left side. On the right side, the typical barcode sign on M-mode is shown.

LUS held optimal accuracy, with superior sensitivity and similar specificity compared to chest X-ray for the diagnosis of pneumothorax.^1,36^ Quite surprisingly, LUS is even more accurate than CT in the evaluation of pneumothorax size.^1,36^ Diagnosis of pneumothorax by LUS is based on the presence of three major findings: (i) absence of lung sliding and lung pulse, (ii) the absence of B lines, and (iii) the ‘lung point’ (e.g. the point where the pleura stops its movement).^1,36^ The first two signs must be present to make a diagnosis, whereas the lung point may not always be detectable. The barcode sign on M-mode is another typical sign of pneumothorax (*Figure 5*).^1,36^ Pneumothorax is a common complication in cardiac surgery; thus, LUS may be extremely useful for its diagnosis and follow-up in this setting.^13,37^ The use of LUS may be of great help to monitor the occurrence of pneumothorax after drainage removal, avoiding serial chest X-ray as is routine practice in many centres^37^ (*Figure 5*; *Video 2*).

##### Key points

- LUS allows for accurate diagnosis of pneumothorax.
- The use of LUS should be encouraged to monitor the occurrence of pneumothorax after drainage removal.

### Complimentary diagnosis and advance use of LUS

#### The retrosternal area: diagnosis of clots and evaluation of retrosternal structures

**Figure 6:**
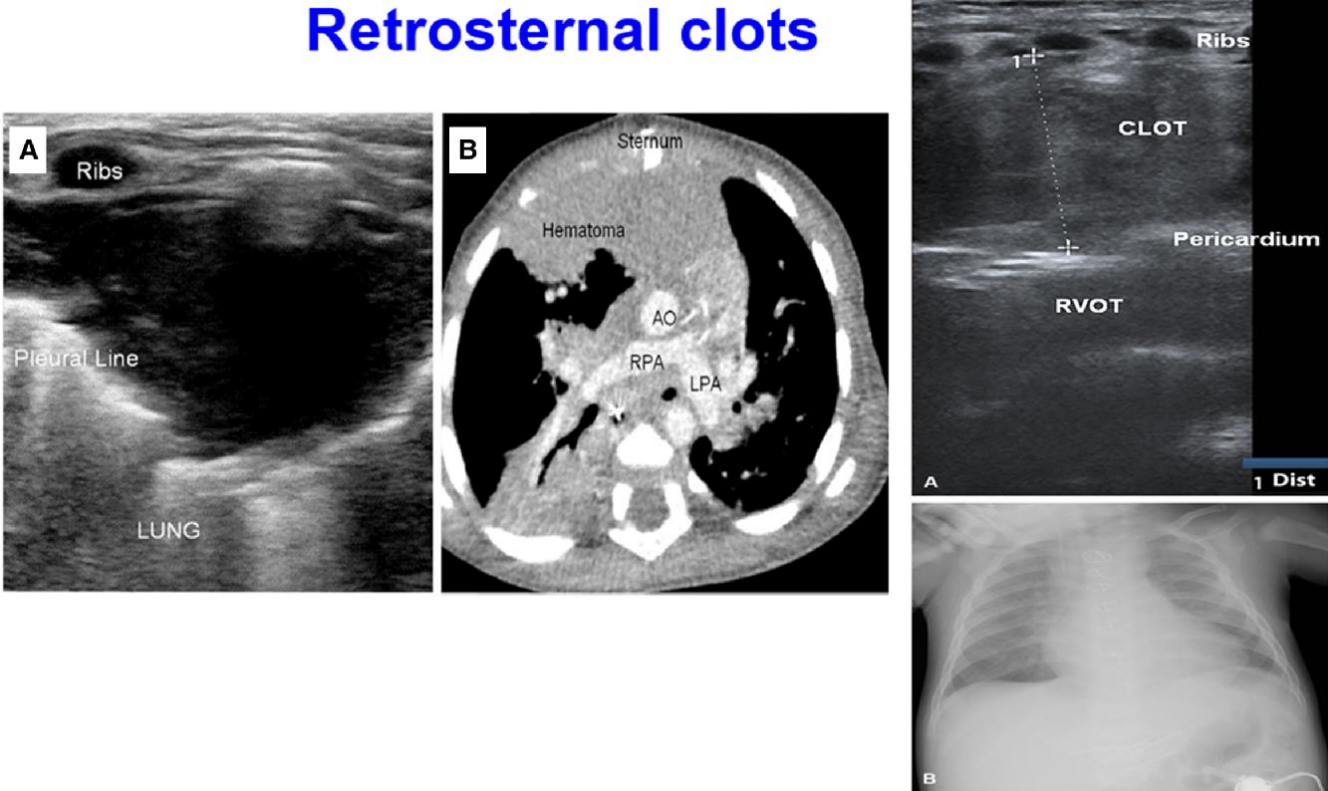
Retrosternal clot. (Left) In (*A*), a retrosternal clot is visualized by LUS and confirmed (*B*) by CT scan. Using LUS, it is possible to appreciate how the clot is interposed among the sternum and the pleural line. (Right) A retrosternal clot among the sternum and the right ventricular outflow tract (RVOT) is visualized by LUS (*A*). On chest X-ray, enlargement of the right mediastinum can be observed.

When performing LUS examination after cardiac surgery, in the scanning of the anterior areas of each hemithorax, it may be useful to explore the para-sternal area.^8,9,29^ Clots after paediatric cardiac surgery in fact are commonly detached close to the sternum. Haematoma and infections (characterized by retrosternal fluid collection and para-sternal hyperconvexity) may also be discovered.^8,9,29^ The probe should be placed close to the sternum, and the anterior area should be scanned up and down and laterally up to the para-sternal line. When a clot, haematoma, or infection is visualized, the probe should be placed over it and freely tilted in various planes or orientations for visualization.^8,9,29^ Patients after total cavo-pulmonary connection are at high risk for development of clots. In a series over 37 children after total cavo-pulmonary connection (mean age 5.5 ± 1.8 years),^8,9^ the presence of retrosternal clots was systematically investigated. Due to the lack of a standardized system to measure and classify clot dimensions, a semiquantitative classification of clot dimension was employed, considering the body size (e.g. mean body surface area 0.7 ± 0.1 m^2^, range 0.3–1.6 m^2^).^8,9^ According to the maximal diameter of the clot on a perpendicular axis, they were classified as large (>3 cm); moderate sized (>2 to <3 cm); small to moderate sized (>1 to <2 cm); and small (<1 cm.).^8,9^ Clots of different sizes were detected in half of the cases and were small in 13.5%, small to moderate in 5.4%, moderate in 8.1%, and large in 16.2%. Just in two cases (two large clots), surgical revision was required^8,9^ (*Figure 6*).

In neonates and infants, furthermore, the use of a linear probe close to the sternum may offer an overview of the entire heart and its relationship with sternal structures and help to better delineate retrosternal structures that may be difficult to visualize with phased-array probes such as right ventricle/systemic ventricle to pulmonary artery conduits^8,9^ (Supplementary data online, *Video S3*).

##### Key points

- Evaluation of retrosternal area during LUS examination is advised to discover retrosternal clots that are common after paediatric cardiac surgery. Haematoma and infection may also be discovered.
- The probe should be placed close to the sternum, and each hemithorax should be scanned up and down and laterally up to the para-sternal line.

##### Gaps of knowledge

- Systems to classify clot severity are currently lacking. Semiquantitative classification of clot dimensions may be performed in relation to body size and chest dimensions (*Table 3*).

#### Diaphragmatic motion anomalies

LUS examination may be used also to evaluate diaphragmatic structure and motion. Diaphragmatic motion may be easily investigated by subcostal view either with LUS or with conventional echocardiography.

**Table 3:** Fundamental elements of LUS examination after paediatric cardiac surgery

	Basic analysis	Advanced examination
B lines	Describe the presence of B lines, their distribution, and perform basic quantification.	Describe characteristics and distinguish B lines due to extravascular fluid accumulation from other forms of de-aeration (e.g. atelectasis, pneumonia, ARDS, and pulmonary fibrosis).
Effusion	Describe the presence and the site of effusion, and perform basic quantification.	Describe characteristics and distinguish among transudate, exudate, haemothorax, and chylothorax.
Atelectasis/consolidations	Describe the presence and the site of consolidation and perform basic quantification.	Describe characteristics and distinguish among atelectasis and other forms of consolidation and masses (e.g. pneumonia, infarction, contusion from thoracic trauma, and primary or metastatic cancer clots).
Pneumothorax	Describe the presence and the site of pneumothorax.	Try to quantify pneumothorax extension.
Retrosternal area	Describe the presence and the site of clots and perform basic quantification.	Describe characteristics and distinguish among clots, haematoma, and infection.

ARDS, acute respiratory distress syndrome.

**Figure 7:**
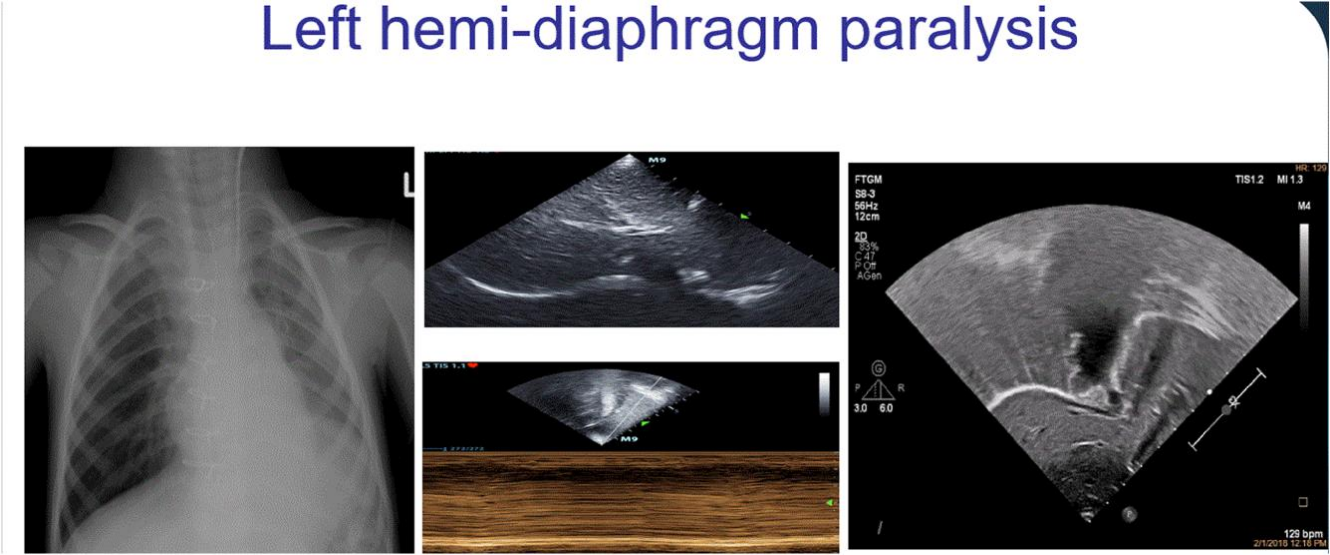
A left hemidiaphragm paralysis can be visualized by chest X-ray (left side) and confirmed by echographic analysis of the diaphragm by subcostal view. In the middle, a paradoxical motion of the diaphragm can be appreciated in M-mode, and on the right image, the left hemidiaphragm lifted can be appreciated (Supplementary data online, *Video S2*).

Diaphragmatic paralysis is a serious and frequent (0.3–12.8% of cases) complication after paediatric cardiac surgery, which may bring to serious pulmonary complications including respiratory insufficiency, pulmonary infections, and cause difficult extubation and prolongation of hospital stay.^29,38^ Diaphragmatic motion may be classified according to their movement towards the probe (by M-mode) and by comparing in subxiphoid view the difference in excursion among the right and left hemidiaphragms.^38,39^ Accordingly, diaphragmatic motion may be classified as (i) normal (e.g. the diaphragm move towards the transducer in inspiration with difference of excursion between the hemidiaphragms is <50%); (ii) decreased (when the difference in the amplitude between the hemidiaphragm is >50%); (iii) absent (typically a flat line at M-mode is visualized); or (iv) paradoxical (absent and paradoxical motion away from the transducer in inspiration) that indicates a diaphragmatic paralysis (usually associated with concomitant atelectasis)^29,38,39^ (*Figure 7*; Video 3).

Furthermore, there is an increased interest in quantification of diaphragmatic movements (e.g. diaphragm thickness, diaphragm excursion, and diaphragm thickening), for monitoring of pulmonary recovery and response to therapy.^40,41^

##### Key points

- Diaphragmatic motion by subcostal view should be systematically investigated after paediatric cardiac surgery (and in those who underwent previous interventions) since they represent a serious and common complication of paediatric cardiac surgery.
- Diaphragmatic motion may be classified as normal, decreased, absent, and paradoxical (e.g. indicating diaphragmatic paralysis) according to their motion in M-mode and by comparison of two hemidiaphragms.

#### LUS guidance of interventional procedures

The use of LUS may serve as a guide and for immediate verification of the result and incidence of complication in a few common interventional procedures, including drainage insertion (for effusion or pneumothorax),^42^ lung recruitment for atelectasis,^43^ and tracheal tube verification during intubation.^44,45^ During thoracentesis, the systematic use of LUS may significantly reduce the incidence of pneumothorax by 8.8–0.97% (*P* < 0.0001) according to adults’ studies.^40^ In neonates, infants, and children,^44,45^ ultrasonography verification of endotracheal tube is feasible (e.g. 83–100%), tolerated, and held a good sensitivity (i.e. 0.91–1.00) and specificity (e.g. 5–1.0).

##### Key points

- The use of LUS is recommended in drainage insertion to guide the procedure and verify the result, and the incidence of complications.
- The use of LUS is helpful in lung recruitment.
- Ultrasonography may help in endotracheal tube verification during intubation.

## Future perspectives

Adult studies suggest that newer speckle tracking echocardiography modalities may be applied to LUS allowing for a better evaluation of lung sliding increasing the accuracy of the diagnosis of pneumothorax^46,47^; however, data in neonates and children are lacking. The use of contrast agents has been proven to be feasible and safe and may serve for the diagnosis of complicated pneumonia (accurately differentiating necrotizing pneumonia from complex parapneumonic effusion)^48^ and visualization of drainage tubes during invasive manoeuvres such as drain insertion. The use of 3D may help in the definition and quantitative/semiquantitative estimation of pulmonary disease. The use of 3D has been limited so far in LUS. In the foetal lung volume, measurement may be accomplished by using either multiplanar technique or a rotational method using VOCAL trademark (Virtual Organ Computer-aided AnaLysis).^49,50^ This application may be particularly helpful for effusion and/or atelectasis quantification; however, irregular 3D geometry of the lungs (figure models) may pose difficulties for accurate mathematical formulas.^49,50^ Recently, teaching and tutorials with interactive 3D models (with the possibility to choose among different types of probes) have been introduced, allowing virtual scanning with different angulations.^51^

## Conclusion and limitations

A guide for the appropriate use of LUS for the diagnosis, management, and follow-up of pulmonary complications in neonates and children with congenital heart disease, with a focus on those after cardiac surgery, has been provided. We advocate that the use of LUS in paediatric cardiology should be promoted and encouraged, since it represents an easy, quick, cheap, radiation-free tool for the diagnosis of common pulmonary complications in neonates and children with CHD after cardiac surgery.

Some knowledge gaps still exist including the difficulty to quantify the size of effusion and atelectasis and to grade their severity. Also, at present, it remains difficult to distinguish among artefacts deriving from water accumulation and other forms of de-aeration.

Further studies are warranted to establish a consensus classification system for the evaluation of disease severity and to expand our knowledge and understanding of artefacts derived by LUS examinations.

## Supplementary Material

qyae134_Supplementary_Data

## Data Availability

No new data were generated or analysed in support of this research.

## Lead author biography

Dr Massimiliano Cantinotti is a Cardiologist at CNR-Tuscany Region Foundation of Massa, and Pisa, Italy. He runs the congenital echocardiography labs. Her has published over 150 per-reviewed papers. His research interest are echocardiography, normal values, lung ultrasound, cardiac biomarkers, and sport cardiology.
