# Supplementary figures and images for: Lung ultrasound in neonates and children with cardiac diseases with focus on post cardiac surgical period: time for systematic use—an expert opinion report by the Association for European Paediatric and Congenital Cardiology Imaging Working Group
Source: Eur Heart J Imaging Methods Pract. 2025 Jan 13;3(1):qyae134. doi: 10.1093/ehjimp/qyae134 (PMC11852288; doi:10.1093/ehjimp/qyae134)

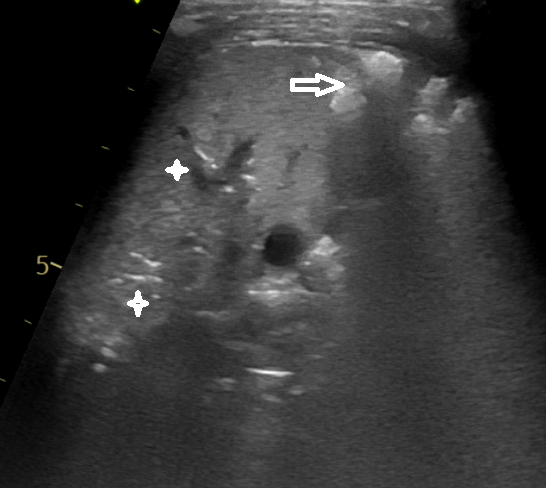

Supplement: qyae134_Supplementary_Data [file qyae134_Supplementary_Data.zip › Supllemntal figure 3.png]

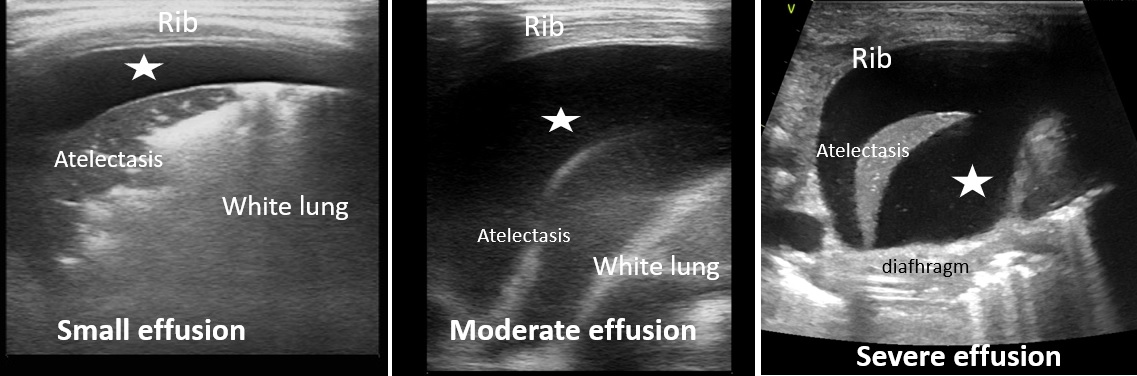

Supplement: qyae134_Supplementary_Data [file qyae134_Supplementary_Data.zip › Supplemental Figure 1..jpg]

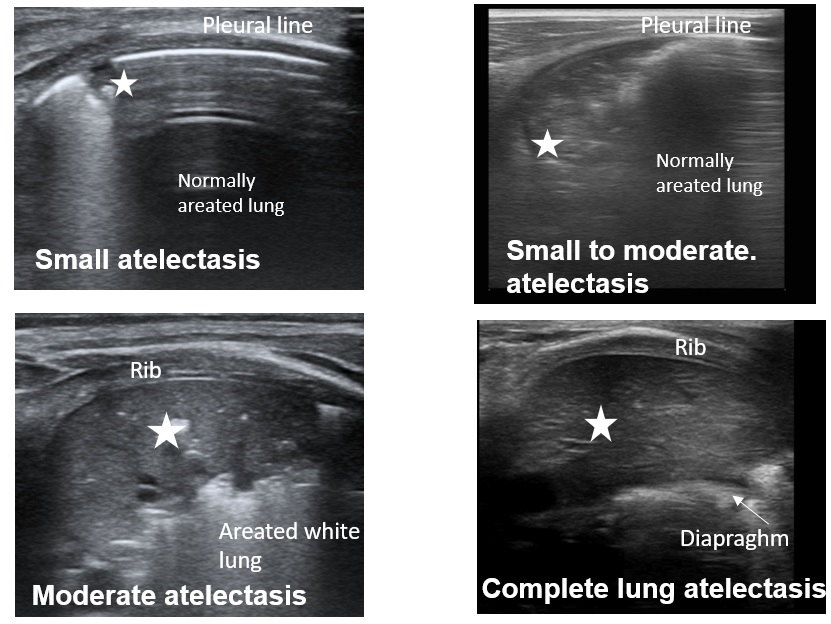

Supplement: qyae134_Supplementary_Data [file qyae134_Supplementary_Data.zip › Supplemental Figure 2.jpg]
